# Decrease in Social Zeitgebers Is Associated With Worsened Delayed Sleep-Wake Phase Disorder: Findings During the Pandemic in Japan

**DOI:** 10.3389/fpsyt.2022.898600

**Published:** 2022-06-09

**Authors:** Rei Otsuki, Kentaro Matsui, Takuya Yoshiike, Kentaro Nagao, Tomohiro Utsumi, Ayumi Tsuru, Naoko Ayabe, Megumi Hazumi, Michio Fukumizu, Kenichi Kuriyama

**Affiliations:** ^1^Department of Laboratory Medicine, National Center Hospital, National Center of Neurology and Psychiatry, Tokyo, Japan; ^2^Department of Sleep-Wake Disorders, National Institute of Mental Health, National Center of Neurology & Psychiatry, Tokyo, Japan; ^3^Department of Psychiatry, Nihon University School of Medicine, Tokyo, Japan; ^4^Department of Psychiatry, National Center Hospital, National Center of Neurology and Psychiatry, Tokyo, Japan; ^5^Department of Psychiatry, The Jikei University School of Medicine, Tokyo, Japan; ^6^Department of Regional Studies and Humanities, Faculty of Education and Human Studies, Akita University, Akita, Japan; ^7^Department of Public Mental Health, National Institute of Mental Health, National Center of Neurology and Psychiatry, Tokyo, Japan; ^8^Segawa Memorial Neurological Clinic for Children, Tokyo, Japan

**Keywords:** delayed sleep-wake phase disorder, coronavirus disease 2019, COVID-19, state of emergency, Japan, social zeitgeber, bipolar disorder, depression

## Abstract

**Background:**

Delay in sleep-wake rhythms was observed in the general population during the coronavirus disease 2019 (COVID-19) pandemic. Patients with delayed sleep-wake phase disorder (DSWPD) may have also experienced exacerbation of symptoms, but no studies have investigated this topic. In this study, we aimed to retrospectively examine the changes in symptoms of outpatients with DSWPD both before and during the pandemic and to identify the factors associated with the exacerbation of sleep-wake rhythms.

**Methods:**

We included outpatients with DSWPD aged 16 years or older who visited the outpatient clinic due to sleep disorders between January and September 2020. Decreased social zeitgebers was defined as a reduction of 50% or more in the frequency of commuting to school or work during the COVID-19 pandemic. The severity of DSWPD was assessed using the clinical global impressions - severity of illness (CGI-S) at two points: before and during the pandemic. We defined the worsened, unchanged, and improved groups as those whose CGI-S scores worsened by at least one point, remained unchanged, and improved by at least one point, respectively. Multivariate logistic regression analysis was performed to determine the factors associated with worsened DSWPD symptoms.

**Results:**

Sixty patients with DSWPD were eligible for this study. Even before the pandemic, patients who were unemployed or did not attend school tended to show more severe DSWPD symptoms. During the pandemic, 27 patients belonged to the worsened group; 28 patients, unchanged group; and 5 patients, improved group. Decreased social zeitgebers (odds ratio [OR] = 6.668, 95% confidence interval [CI]: 1.653–26.891, *p* < 0.05) and comorbid mood disorders (OR = 8.876, 95% CI: 1.714–45.974, *p* < 0.05) showed independent significant associations with the worsening of DSWPD symptoms.

**Conclusions:**

During the pandemic, the symptoms of DSWPD tended to worsen. The obtained findings emphasize the importance of social zeitgebers, suggesting the need for external motivation in DSWPD treatment.

## Introduction

Circadian rhythm sleep-wake disorders (CRSWDs) are characterized by an inability to synchronize the endogenous circadian rhythm with the daily sleep-wake schedule required for social life, resulting in distress and social disadvantage for the individual (1). CRSWDs consist of several disorders, and delayed sleep-wake phase disorder (DSWPD) is the most common. The prevalence of DSWPD in the general population ranges from 0.1 to 5.1% (2–4), and a higher prevalence (1.1 to 15.9%) has been reported in adolescents and young adults (5–7). DSWPD is known to have serious negative social consequences due to pronounced difficulty falling asleep and waking up at the desired time. These symptoms result in difficulty going to work or school tardiness and absence, as well as falling asleep or poor performance during working hours, with mental health problems, including anxiety and depression (8–11). DSWPD is often comorbid with mood disorders, schizophrenia, and developmental disorders. Moreover, it has been suggested that these comorbidities are associated with worsened symptoms of their own disorders (12–15). DSWPD comorbid with psychiatric or developmental disorders has been reported to have different clinical characteristics compared with primary DSWPD, such as older age, higher rates of unemployment, and poorer treatment responsiveness (10, 16).

Since the intrinsic cycle of the human circadian rhythm is slightly longer than 24 h (17–20), synchronizing factors that advance the circadian phase are considered essential to keep the sleep-wake rhythms synchronized to 24 h. Not only by exposure to high-intensity light immediately after waking (21), non-photic social zeitgebers, such as work and school during the daytime, contribute to preventing delayed sleep-wake phase rhythms (22). However, some people have been in confinement due to the coronavirus disease (COVID-19)-related lockdown, which may have disrupted their sleep-wake rhythms due to the loss of social zeitgebers (23). Delay in bed time and waking time of workers and students as a result of the lockdown has already been reported (24). A tendency toward delayed sleep-wake rhythms during the pandemic has also been indicated in the general population (25–28); thus, the possibility of increased DSWPD development has been pointed out (29).

Patients with DSWPD who are already receiving treatment may also have experienced a delay in their sleep-wake rhythms during the pandemic. Although the vulnerability to circadian rhythm dysregulation in patients with DSWPD has been widely reported (30–32), the association of decreased social zeitgebers during the pandemic with changes in the sleep-wake schedule in patients with DSWPD has not yet been elucidated. Furthermore, no study has investigated how comorbidity with psychiatric or developmental disorders has affected sleep-wake schedules in patients with DSWPD during the COVID-19 pandemic. We hypothesized that worsened DSWPD symptoms would be more pronounced in patients who experienced a significant change in lifestyle, particularly the loss of social zeitgebers due to suspension of commuting to school or work during the pandemic. We also speculated that patients with comorbid psychiatric and developmental disorders would be more likely to experience worsened DSWPD symptoms than those without such disorders, considering their possible vulnerability during the pandemic. We aimed to investigate the changes in the symptoms of DSWPD in outpatients before and during the COVID-19 pandemic to examine the aforementioned hypothesis, as well as to identify the factors associated with the changes in severity among these patients.

## Methods

### Participants

We retrospectively reviewed the medical records of patients with DSWPD aged 16 years or older who visited the outpatient clinic of the National Center Hospital of Neurology and Psychiatry for sleep disorders between January and September 2020. Patients with DSWPD who stopped visiting the hospital or had insufficient information were excluded from the analysis. DSWPD diagnosis was confirmed by board-certified sleep medicine physicians of the Japanese Society of Sleep Research (KM, TY, AT, MF, and KK) according to the International Classification of Sleep Disorders, 3rd edition (1).

### Measurements

The following demographic and clinical data were retrospectively collected from the medical records: age, sex, body mass index (BMI), school/work status, presence of cohabitants, comorbidity of psychiatric disorders and sleep disorders (except DSWPD), introduction of chronobiological interventions including melatonin agonists (ramelteon and melatonin) and bright light therapy (33), and pharmacological treatment using psychotropic medications other than melatonin agonists. The introduction of chronobiological interventions was investigated at baseline and during the pandemic. However, for psychotropic medications other than melatonin agonists, only the baseline status was assessed due to insufficient information in the medical records. For social zeitgebers, we assessed the frequency of commuting to school and work before and during the COVID-19 pandemic and defined decreased social zeitgeber as a reduction of 50% or more in the frequency of commuting to school or work under the COVID-19 pandemic than before.

### Severity and Change in DSWPD Symptoms

The severity of DSWPD symptoms was retrospectively evaluated using the clinical global impressions - severity of illness (CGI-S) (34) for two periods: baseline, from January to March 2020 (before the COVID-19 pandemic), and endpoint, from April to September 2020 (during the COVID-19 pandemic). CGI-S was scored as follows: 1 = normal, not sick at all; 2 = borderline psychosis; 3 = mild illness; 4 = moderate illness; 5 = marked illness; 6 = severe illness; and 7 = most severe illness. We used the CGI-S because there has been no internationally accepted severity scale for DSWPD. In addition, the CGI-S is simple, can be examined retrospectively using the same criteria, and has been used to rate the severity of DSWPD in some previous studies (35, 36). When determining the CGI-S score, two board-certified psychiatrists of the Japanese Society of Psychiatry and Neurology (RO and KM, KM is also a board-certified sleep medicine physician of the Japanese Society of Sleep Research) evaluated the severity of DSWPD, according to the difference between the desired time and the actual time of sleep onset and awakening and the percentage of days when the patient could fall asleep and wake up at the desired time. In some patients for whom sleep logs and actigraphs were recorded, those results were also accounted. Using the median value of the baseline CGI-S scores, we defined a baseline CGI-S score of 4 or higher as moderate-to-severe DSWPD and a baseline CGI-S score of less than four as mild DSWPD. For the change in symptoms during the pandemic, we used the difference in CGI-S scores between baseline and endpoint: an increase of one or more points as worsened, no change as unchanged, and a decrease of one or more points as improved.

### Statistical Analysis

Based on baseline severity, a comparison between mild DSWPD and moderate-to-severe DSWPD was made using the χ^2^ test for categorical variables and the Mann–Whitney *U*-test for the following continuous variables: age, sex, BMI, being a student, presence of cohabitants, comorbidity of psychiatric disorders, comorbidity of sleep disorders other than DSWPD, introduction of chronobiological interventions before the pandemic, and use of psychotropic medications other than melatonin agonists. To investigate the factors associated with worsened DSWPD, logistic regression analysis was conducted for age, sex, BMI, student (yes/no), cohabitation (yes/no), coexistence of psychiatric disorders (schizophrenia/mood disorders/anxiety disorders/intellectual disability, and developmental disorders), coexistence of sleep disorders other than DSWPD (yes/no), and introduction of chronobiological interventions during the COVID-19 pandemic (yes/no). Multiple regression analysis was performed with worsened DSWPD symptoms (i.e., increase of one or more points in CGI-S score) as the dependent variable. All variables were first examined in a univariate model. Then, a multiple regression model was performed on all variables that showed significant correlation in the univariate model to determine the main correlations, controlling for confounding factors. SPSS version 27.0J (SPSS Japan, Inc., Tokyo, Japan) was used for statistical analysis, and the statistical significance level was set at less than 5%.

## Results

Of the 108 patients with DSWPD, 48 were excluded because they had interrupted outpatient visits (*n* = 15), were referred to other outpatient clinics (*n* = 3), and could not be rated for severity due to insufficient information (*n* = 30). Finally, 60 patients with DSWPD were included in the analysis. Table 1 shows the demographic and clinical data of the study subjects. Accordingly, the median age of the total cohort (range) was 24 (16–71) years, and 56.7% of the subjects were male. The median baseline CGI-S score (range) was 3 (2–6), and the median endpoint CGI-S score (range) was 4 (2–6). A total of 26 patients (43.3%) were students, and 20 patients (33.3%) were unemployed or did not attend school at baseline. Notably, 38 patients (63.3%) experienced decreased social zeitgebers during the COVID-19 pandemic. Psychiatric or developmental disorders were comorbid in 26 patients (43.3%): 1 had schizophrenia, 13 had mood disorders (including 9 with major depressive disorder and 4 with bipolar disorder), 6 had anxiety disorders (including 1 with generalized anxiety disorder, 3 with social anxiety disorder, and 2 with obsessive-compulsive disorder), and 11 had developmental disorders (including 2 with attention-deficit/hyperactivity disorder, 8 with autism spectrum disorder, and 1 with both, with some overlap to other psychiatric disorders). The number of patients with sleep disorders other than DSWPD was 18 (30%), including 12 with obstructive sleep apnea, 3 with central hypersomnia (including 2 with narcolepsy type 2, 1 with idiopathic hypersomnia), 3 with sleep-related movement disorders (including 1 with restless legs syndrome and 2 with periodic limb movement disorder), and 1 with parasomnia (sleep-related eating disorder). Forty-four (73.3%) patients received chronobiological interventions and 33 (55%) took psychotropic medications at baseline.

**Table 1:** Demographic and clinical data of patients with DSWPD (*n* = 60).

Age, median (range), year	24.0 (16–71)
Male, *n* (%)	34 (56.7)
BMI, median (range), kg/cm^2^	20.5 (15.9–32.3)
Student, *n* (%)	26 (43.3)
Unemployed or did not attend school, *n* (%)	20 (33.3)
Cohabitation, *n* (%)	43 (71.7)
Decreased social zeitgebers, *n* (%)	38 (63.3)
Baseline CGI-S, median (range)^a^	3.0 (2.0–6.0)
Endpoint CGI-S, median (range)^b^	4.0 (2.0–6.0)
**Coexisting mental disorders**
Schizophrenia, *n* (%)	1 (1.7)
Mood disorders, *n* (%)^c^	13 (21.7)
Anxiety disorders, *n* (%)^d^	6 (10.0)
Developmental disorders, *n* (%)^e^	11 (18.3)
**Other coexisting sleep disorders**
Obstructive sleep apnea, *n* (%)	12 (20.0)
Central disorders of hypersomnolence, *n* (%)^f^	3 (5.0)
Sleep-related movement disorders, *n* (%)^g^	3 (5.0)
Parasomnias, *n* (%)^h^	1 (1.7)
Chronobiological intervention at baseline^i^	44 (73.3)
**Psychotropic medication other than ramelteon and melatonin**
Antipsychotics, *n* (%)	15 (25.0)
Antidepressants, *n* (%)	12 (20.0)
Mood stabilizers, *n* (%)	6 (10.0)
Benzodiazepines, *n* (%)	12 (20.0)
Non-benzodiazepines, *n* (%)	7 (11.7)
Orexin receptor antagonists, *n* (%)	8 (13.3)
Psychostimulants, *n* (%)	5 (8.3)

a Baseline CGI-S: before the COVID-19 pandemic.

b Endpoint CGI-S: during the COVID-19 pandemic.

c Including major depressive disorder (*n* = 9) and bipolar disorder (*n* = 4).

d Including generalized anxiety disorder (*n* = 1), social anxiety disorder (*n* = 3), and obsessive-compulsive disorder (*n* = 2).

e Including attention-deficit/hyperactivity disorder (*n* = 2), autism spectrum disorder (*n* = 8), and a combination of both (*n* = 1).

f Including narcolepsy type two (*n* = 2) and idiopathic hypersomnia (*n* = 1).

g Including restless legs (*n* = 1) and periodic limb movements (*n* = 2).

h Sleep-related eating disorder.

i Including use of ramelteon or melatonin (*n* = 37), bright light therapy (*n* = 1), and combined therapy with both (*n* = 6).

DSWPD, delayed sleep-wake phase disorder; BMI, body mass index; CGI-S, clinical global impressions - severity of illness scale.

Before the pandemic, 32 and 28 patients were considered to have mild DSWPD and moderate-to-severe DSWPD, respectively, according to the baseline CGI-S score. Comparison between the two groups showed that the moderate-to-severe DSWPD group had significantly more patients who were unemployed or did not attend school (*p* = 0.001) than the mild DSWPD group. The patients in the mild DSWPD group tended to be older, have a higher BMI, and were more likely to have comorbidities of other sleep disorders than moderate-to-severe DSWPD, but these differences were not significant (*p* = 0.061, *p* = 0.064, and *p* = 0.055, respectively) (Table 2).

**Table 2:** Characteristics of patients before the pandemic: mild vs. moderate-to-severe DSWPD.

	**Mild DSWPD (*n* = 32)^a^**	**Moderate-to-severe DSWPD** **(*n* = 28)^a^**	***p*** **^b^**
Age, median (range), year	27.5 (16–71)	20.5 (16.0–58.0)	0.061
Male, *n* (%)	19 (59.4)	15 (53.6)	0.651
BMI, median (range), kg/cm^2^	21.2 (17.0–32.3)	19.3 (15.9–30.3)	0.064
Student, *n* (%)	11 (34.4)	15 (53.6)	0.134
Unemployed or did not attend school, *n* (%)	6 (18.8)	14 (50.0)	0.010
Cohabitation, *n* (%)	22 (68.8)	21 (75.0)	0.592
Coexisting mental disorders	15 (46.9)	10 (35.7)	0.382
Schizophrenia, *n* (%)	1 (3.1)	0	0.346
Mood disorders, *n* (%)	8 (25.0)	5 (17.9)	0.503
Anxiety disorders, *n* (%)	2 (6.3)	4 (14.3)	0.301
Developmental disorders, *n* (%)	7 (21.9)	4 (14.3)	0.448
Other coexisting sleep disorders	13 (40.6)	5 (17.9)	0.055
Chronobiological intervention at baseline	21 (65.6)	23 (82.1)	0.149
Psychotropic medications other than ramelteon and melatonin	17 (53.1)	16 (57.1)	0.755

a *Using the median value of the baseline CGI-S scores, we defined a baseline CGI-S score of four or higher as moderate-to-severe DSWPD and a baseline CGI-S score of less than four as mild*.

b *Chi-square test (categorical variables) or Mann-Whitney U-test (continuous variables)*.

*DSWPD, delayed sleep-wake phase disorder; BMI, body mass index; CGI-S, clinical global impressions - severity of illness scale*.

Baseline and endpoint CGI-S scores showed that 27 patients worsened, 28 remained unchanged, and 5 improved with regard to DSWPD symptoms. For patients with worsened DSWPD symptoms, univariate logistic regression analysis showed that decreased social zeitgebers (odds ratio [OR] = 4.675, 95% confidence interval [CI]: 1.427–15.321, *p* < 0.05) and comorbidity of mood disorders (OR = 5.882, 95% CI: 1.421–24.355, *p* < 0.05) were significantly associated. In the multiple logistic regression model, both decreased social zeitgebers (OR = 6.668, 95% CI: 1.653–26.891, *p* < 0.05) and comorbidity of mood disorders (OR = 8.876, 95% CI: 1.714–45.974, *p* < 0.05) exhibited independent significant associations (Table 3).

**Table 3:** Factors associated with worsened DSWPD symptoms^a^.

	**Univariate relative risk (95% CI)^b^**	***p***	**Multivariate relative risk (95% CI)^b^**	***p***
Age	1.004 (0.965–1.045)	n.s.		
Male sex	0.700 (0.250–1.957)	n.s.		
BMI	1.072 (0.929–1.238)	n.s.		
Student	1.429 (0.511–3.995)	n.s.		
Unemployed or did not attend school	0.388 (0.124–1.212)	n.s.		
Cohabitation	2.514 (0.755–8.368)	n.s.		
Decreased social zeitgebers	4.675 (1.427–15.321)	0.011	6.668 (1.653–26.891)	0.008
**Coexisting mental disorders**
Schizophrenia	—	—		
Mood disorders	5.882 (1.421–24.355)	0.015	8.876 (1.714–45.974)	0.009
Anxiety disorders	1.250 (0.231–6.760)	n.s.		
Developmental disorders	0.646 (0.167–2.493)	n.s.		
Other coexisting sleep disorders^c^	0.968 (0.319–2.940)	n.s.		
Chronobiological intervention during the pandemic^d^	1.242 (0.399–3.871)	n.s.		
Psychotropic medications other than ramelteon and melatonin	2.400 (0.837–6.882)	n.s.		

a Using the difference in CGI-S scores before and during the COVID-19 pandemic, we defined the worsened group as one or more points increase.

b Relative risks approximated to odds ratios.

c Including obstructive sleep apnea (*n* = 11), central disorders of hypersomnolence (*n* = 3), sleep-related movement disorders (*n* = 2), parasomnias (*n* = 1), and combination of obstructive sleep apnea with sleep-related movement disorders (*n* = 1).

d Including use of ramelteon or melatonin (*n* = 36), bright light therapy (*n* = 1), and combination therapy of both (*n* = 6).

During the observation period, only 1 out of the 44 patients discontinued chronobiological interventions. A total of 43 patients received chronobiological interventions during the pandemic: 36 patients received melatonin agonists (melatonin, ramelteon) only, 1 patient received bright light therapy only, and 6 patients received combination therapy of melatonin agonists and bright light therapy. The use of melatonin agonists, bright light therapy, and combinations of these two therapies in these three groups are shown separately in the mild and moderate-to-severe DSWPD groups (Table 4). All five patients in the improvement group received chronobiological interventions (four, ramelteon or melatonin only; one, combination therapy with melatonin agonists and bright light therapy). Even under treatment, 14 of the 20 patients in the mild DSWPD group worsened. Meanwhile, the majority of the patients in the moderate-to-severe DSWPD group showed no change in symptoms.

**Table 4:** Breakdown of chronobiological intervention during the COVID-19 pandemic, baseline severity of DSWPD, and changes in symptoms.

	**Worsened^**b**^(*n* = 27)**	**Unchanged^**b**^(*n* = 28)**	**Improved^**b**^(*n* = 5)**
**Use of ramelteon or melatonin**			
Mild^a^	13	5	1
Moderate-to-severe^a^	4	10	3
**Bright light therapy**			
Mild^a^	1	0	0
Moderate-to-severe^a^	0	0	0
**Combination therapy of both**			
Mild^a^	0	0	0
Moderate-to-severe^a^	2	3	1
**No chronobiological intervention**			
Mild^a^	6	6	0
Moderate-to-severe^a^	1	4	0

a *Using the median value of the baseline CGI-S scores, we defined a baseline CGI-S score of four or higher as moderate-to-severe DSWPD and a baseline CGI-S score of less than four as mild DSWPD*.

b *Using the difference in CGI-S scores before and during the COVID-19 pandemic, we defined the worsened group as one or more points increase, improved group as one or more points decrease, and unchanged group as no change. DSWPD, delayed sleep-wake phase disorder; CGI-S, clinical global impressions - severity of illness scale*.

## Discussion

The present study examined the changes in severity among patients with DSWPD symptoms during the COVID-19 pandemic. As we hypothesized, reduced social zeitgeber showed an association with worsened severity of DSWPD. Environmental, social, and behavioral factors have already been identified as potentially important in DSWPD treatment (37, 38). However, to the best of our knowledge, this study is the first to address the possible effect of social zeitgebers on symptom severity and course of treatment in DSWPD, even when reviewing the literature prior to the COVID-19 pandemic.

Although bright light exposure in the morning advances the sleep-wake phase and stabilizes sleep-wake rhythms (21), whether the loss of social zeitgebers resulted in delayed sleep-wake rhythms *via* reduced opportunities for morning light exposure or whether it simply reduced the motivation to wake up in the morning, regardless of light exposure, remains unclear. However, regardless of whether bright light therapy was demonstrated, hospitalization is effective in modulating the sleep-wake rhythms of patients with CRSWD, including DSWPD (39), suggesting the importance of enforcement to wake up in DSWPD treatment. In addition, the moderate-to-severe DSWPD group had more patients who were unemployed or did not attend school than the mild DSWPD group at baseline in the present study. This finding suggests that even before the COVID-19 pandemic, the lack of social zeitgebers may have been associated with the severity of DSWPD or poor response to the intervention. However, poor response to treatment itself might have led to a decrease in social zeitgebers; thus, the causal relationship between the two remains unclear. Recent case reports of DSWPD by Epstein and colleagues indicated that being free from work during the pandemic had delayed sleep rhythms but improved sleep regularity and daytime sleepiness (40). For the two cases presented by them, environmental adjustments that allow the patient to work with a delayed sleep-wake rhythm would be more desirable than enforcing a morning-type sleep-wake rhythm. In the treatment of DSWPD, the main focus has been on advancing the sleep-wake rhythm to meet social needs (33), and the present study suggests that the introduction of factors that motivate or enforce getting up may be effective in this regard. However, given the findings that DSWPD or extreme evening chronotype can be derived from genetic vulnerability (41–44), another goal of treatment, i.e., to improve daily functioning, including employment, while the sleep-wake rhythm remains delayed, can also be an option. Future studies should examine this proposed goal setting in DSWPD treatment, as there is currently no consensus on this methodology that is not necessarily aimed at modifying the sleep-wake rhythm.

Comorbid mood disorders were significantly associated with worsened DSWPD symptoms independently from decreased social zeitgebers. Comorbidity of DSWPD to mood disorders is common; moreover, it has been suggested that patients with mood disorders are vulnerable to the disruption of sleep-wake schedules (45, 46). During the COVID-19 pandemic, symptoms of depression and anxiety in patients with major depressive disorder and bipolar disorder worsened (47, 48). Particularly for bipolar disorder, CRSWD is considered to be closely related to its pathogenesis (49); lockdown may have led to the recurrence of depressive symptoms in patients with bipolar disorder through the dysregulation of sleep-wake rhythms (50). Considering that psychiatric symptoms, such as anxiety and depression, are known to cause disruptions in sleep-wake rhythms (13), such co-occurrence of exacerbation in mood symptoms and sleep-wake rhythm dysregulations during the pandemic may have occurred not only in patients with bipolar disorder but also in those with mood disorders in general. The subjects in the present study may have also had worsened DSWPD symptoms combined with exacerbation of mood episodes. However, due to the lack of information on changes in the severity of psychiatric symptoms, we could not examine the association between mood episodes and sleep-wake rhythm disorders. To evaluate the relationship between mood symptoms and sleep-wake rhythms, future studies should investigate the severity of mood symptoms as well as changes in sleep-wake rhythms.

Although chronobiological treatments, including bright light therapy and pharmacological treatment with melatonin agonists, have been recognized as effective for sleep-wake dysregulation in patients with DSWPD (51–54), the protective effect of chronobiological interventions on the exacerbation of DSWPD symptoms during the pandemic was not suggested in the present study. In this study, 23 out of the 28 patients in the moderate-to-severe DSWPD group had already received chronobiological interventions before the COVID-19 pandemic. This result suggests that a certain subset of patients with DSWPD does not respond well to the treatments. Moreover, 14 of the 21 patients in the mild DSWPD group who received chronobiological interventions also had worsening of DSWPD symptoms during the pandemic. These results indicate that the maintenance of social synchronization factors may be a prerequisite for the effectiveness of chronobiological interventions. However, of note, all of the five patients who improved during the COVID-19 pandemic (including three patients with decreased social zeitgebers) received chronobiological interventions. Therefore, although its efficacy rate may not be high, the use of melatonin agonists or bright light therapy could still be beneficial in patients with DSWPD. To date, the treatment of DSWPD, particularly interventions for refractory cases, has not been well established (33). Therefore, the development and evaluation of efficacious chronobiological interventions are warranted in future research.

This study had several limitations that should be addressed. First, it was a retrospective study at a single institution that has a specialized outpatient clinic for sleep disorders. Furthermore, only the CGI-S was used to assess severity, and the detailed records of sleep habits were not examined. We also did not use the CGI-I score because we had limited medical record information, and it was difficult to assess the level of improvement in detail on a seven-point scale. In addition, a multinomial logistic analysis using the unchanged group as a reference would have been preferable given that some of the subjects had improved in their DSWPD symptoms. However, we did not employ that analysis in the present study due to the small sample size. Second, changes in sleep-wake rhythms during the lockdown could be explained by several factors, including increased screen exposure time, loss of regularity in dietary rhythms, decreased physical activity, and changes in daytime light exposure (55). In particular, exposure to blue light can delay the sleep onset latency (25, 56); yet, the effect of increased exposure to blue light due to longer screen time during the pandemic (57) was not examined in this study. Future studies should comprehensively investigate the living conditions potentially affecting sleep-wake rhythms. Third, the levels of psychiatric and physical symptoms were not assessed. Furthermore, although various stressors due to the pandemic may have affected sleep-wake rhythms, either directly or through depressive symptoms, these stress factors were not controlled for in this study.

## Conclusion

The symptoms of DSWPD tended to worsen during the pandemic. The decrease in social zeitgebers following the COVID-19 pandemic was suggested to be a major factor in the exacerbation of symptoms in patients with DSWPD. The importance of social zeitgebers, i.e., external enforcement to waking up, has been emphasized; it may have been linked to stabilizing sleep-wake rhythms more than the treatments that are conventionally considered effective for DSWPD. Hence, the active implementation of social zeitgebers could be a novel intervention in DSWPD treatment. Further studies should clarify the impact of patient characteristics or mood symptoms on therapeutic response and the effectiveness of external enforcement in DSWPD treatment.

## Data Availability Statement

The clinical datasets of participants presented in this article are not readily available due to ethical reasons. Requests to access the datasets should be directed to KM, matsui.kentaro@ncnp.go.jp.

## Ethics Statement

This study was approved by the Ethics Committee of the National Center for Neurology and Psychiatry Hospital (A2020-092). Written informed consent from the participants' legal guardian/next of kin was not required to participate in this study in accordance with the National Legislation and the Institutional Requirements.

## Author Contributions

RO, KM, TY, KN, TU, AT, NA, MH, MF, and KK contributed to the study design, data collection, result interpretation, and manuscript preparation. RO and KM performed the statistical analysis of the data. RO, KM, and KK drafted the manuscript. All authors contributed to the article and approved the submitted version.

## Funding

This study was supported by JSPS KAKENHI Grant-in-Aid for Young Scientists (Grant No. 19K17098).

## Conflict of Interest

The authors declare that the research was conducted in the absence of any commercial or financial relationships that could be construed as a potential conflict of interest.

## Publisher's Note

All claims expressed in this article are solely those of the authors and do not necessarily represent those of their affiliated organizations, or those of the publisher, the editors and the reviewers. Any product that may be evaluated in this article, or claim that may be made by its manufacturer, is not guaranteed or endorsed by the publisher.
