# Corrigendum: Decrease in social zeitgebers is associated with worsened delayed sleep-wake phase disorder: findings during the pandemic in Japan

**DOI:** 10.3389/fpsyt.2023.1238041

**Published:** 2023-07-04

**Authors:** Rei Otsuki, Kentaro Matsui, Takuya Yoshiike, Kentaro Nagao, Tomohiro Utsumi, Ayumi Tsuru, Naoko Ayabe, Megumi Hazumi, Michio Fukumizu, Kenichi Kuriyama

**Affiliations:** ^1^Department of Laboratory Medicine, National Center Hospital, National Center of Neurology and Psychiatry, Tokyo, Japan; ^2^Department of Sleep-Wake Disorders, National Institute of Mental Health, National Center of Neurology & Psychiatry, Tokyo, Japan; ^3^Department of Psychiatry, Nihon University School of Medicine, Tokyo, Japan; ^4^Department of Psychiatry, National Center Hospital, National Center of Neurology and Psychiatry, Tokyo, Japan; ^5^Department of Psychiatry, The Jikei University School of Medicine, Tokyo, Japan; ^6^Department of Regional Studies and Humanities, Faculty of Education and Human Studies, Akita University, Akita, Japan; ^7^Department of Public Mental Health, National Institute of Mental Health, National Center of Neurology and Psychiatry, Tokyo, Japan; ^8^Segawa Memorial Neurological Clinic for Children, Tokyo, Japan

**Keywords:** delayed sleep-wake phase disorder, coronavirus disease 2019, COVID-19, state of emergency, Japan, social zeitgeber, bipolar disorder, depression

In the published article, there was an error in Table 1 as published. The entry for Developmental disorders, originally listed as 11 (21.7), is incorrect; the correct figure is Developmental disorders, 11 (18.3). Similarly, the entry for Psychostimulants, previously stated as 5 (7.4), is incorrect; the accurate figure is Psychostimulants, 5 (8.3). The entry for combined therapy with both (*n* = 5) is also incorrect; the correct number is combined therapy with both (*n* = 6). Finally, the entry for Antipsychotics was listed as 15 (25) without decimal point, but the correct version is Antipsychotics, 15 (25.0). The corrected Table 1 and its caption appear below.

**Table 1:** Demographic and clinical data of patients with DSWPD (*n* = 60).

Age, median (range), year	24.0 (16–71)
Male, *n* (%)	34 (56.7)
BMI, median (range), kg/cm^2^	20.5 (15.9–32.3)
Student, *n* (%)	26 (43.3)
Unemployed or did not attend school, *n* (%)	20 (33.3)
Cohabitation, *n* (%)	43 (71.7)
Decreased social zeitgebers, *n* (%)	38 (63.3)
Baseline CGI-S, median (range)^a^	3.0 (2.0–6.0)
Endpoint CGI-S, median (range)^b^	4.0 (2.0–6.0)
**Coexisting mental disorders**	
Schizophrenia, *n* (%)	1 (1.7)
Mood disorders, *n* (%)^c^	13 (21.7)
Anxiety disorders, *n* (%)^d^	6 (10.0)
Developmental disorders, *n* (%)^e^	11 (18.3)
**Other coexisting sleep disorders**	
Obstructive sleep apnea, *n* (%)	12 (20.0)
Central disorders of hypersomnolence, *n* (%)^f^	3 (5.0)
Sleep-related movement disorders, *n* (%)^g^	3 (5.0)
Parasomnias, *n* (%)^h^	1 (1.7)
Chronobiological intervention at baseline^i^	44 (73.3)
**Psychotropic medication other than ramelteon and melatonin**	
Antipsychotics, *n* (%)	15 (25.0)
Antidepressants, *n* (%)	12 (20.0)
Mood stabilizers, *n* (%)	6 (10.0)
Benzodiazepines, *n* (%)	12 (20.0)
Non-benzodiazepines, *n* (%)	7 (11.7)
Orexin receptor antagonists, *n* (%)	8 (13.3)
Psychostimulants, *n* (%)	5 (8.3)

a Baseline CGI-S: before the COVID-19 pandemic.

b Endpoint CGI-S: during the COVID-19 pandemic.

c Including major depressive disorder (*n* = 9) and bipolar disorder (*n* = 4).

d Including generalized anxiety disorder (*n* = 1), social anxiety disorder (*n* = 3), and obsessive-compulsive disorder (*n* = 2).

e Including attention-deficit/hyperactivity disorder (*n* = 2), autism spectrum disorder (*n* = 8), and a combination of both (*n* = 1).

f Including narcolepsy type two (*n* = 2) and idiopathic hypersomnia (*n* = 1).

g Including restless legs (*n* = 1) and periodic limb movements (*n* = 2).

h Sleep-related eating disorder.

i Including use of ramelteon or melatonin (*n* = 37), bright light therapy (*n* = 1), and combined therapy with both (*n* = 6).

DSWPD, delayed sleep-wake phase disorder; BMI, body mass index; CGI-S, clinical global impressions - severity of illness scale.

In the published article, there was an error in Table 3 as published. Within the footnotes, the entry for combination therapy of both, originally listed as (*n* = 5), is incorrect; the correct number is combination therapy of both (*n* = 6). Additionally, the footnote c) was missing the information about the combination of obstructive sleep apnea with sleep-related movement disorders, which should have been mentioned as (*n* = 1). The corrected Table 3 and its caption appear below.

**Table 3:** Factors associated with worsened DSWPD symptoms^a^.

	**Univariate relative risk (95% CI)^b^**	***p***	**Multivariate relative risk (95% CI)^b^**	***p***
Age	1.004 (0.965–1.045)	n.s.		
Male sex	0.700 (0.250–1.957)	n.s.		
BMI	1.072 (0.929–1.238)	n.s.		
Student	1.429 (0.511–3.995)	n.s.		
Unemployed or did not attend school	0.388 (0.124–1.212)	n.s.		
Cohabitation	2.514 (0.755–8.368)	n.s.		
Decreased social zeitgebers	4.675 (1.427–15.321)	0.011	6.668 (1.653–26.891)	0.008
**Coexisting mental disorders**				
Schizophrenia	—	—		
Mood disorders	5.882 (1.421–24.355)	0.015	8.876 (1.714–45.974)	0.009
Anxiety disorders	1.250 (0.231–6.760)	n.s.		
Developmental disorders	0.646 (0.167–2.493)	n.s.		
Other coexisting sleep disorders^c^	0.968 (0.319–2.940)	n.s.		
Chronobiological intervention during the pandemic^d^	1.242 (0.399–3.871)	n.s.		
Psychotropic medications other than ramelteon and melatonin	2.400 (0.837–6.882)	n.s.		

a Using the difference in CGI-S scores before and during the COVID-19 pandemic, we defined the worsened group as one or more points increase.

b Relative risks approximated to odds ratios.

c Including obstructive sleep apnea (*n* = 11), central disorders of hypersomnolence (*n* = 3), sleep-related movement disorders (*n* = 2), parasomnias (*n* = 1), and combination of obstructive sleep apnea with sleep-related movement disorders (*n* = 1).

d Including use of ramelteon or melatonin (*n* = 36), bright light therapy (*n* = 1), and combination therapy of both (*n* = 6).

In the published article, there was also an error in the text. The term “?^2^ test” was incorrect, and the correct term is the “χ^2^ test”.

An amendment has been made in the Methods section, specifically the Statistical analysis part, on Line 2.

Originally, the sentence stated:

“Based on baseline severity, a comparison between mild DSWPD and moderate-to-severe DSWPD was made using the ?^2^ test for categorical variables and the Mann-Whitney U-test for the following continuous variables:”

The corrected sentence appears below:

“Based on baseline severity, a comparison between mild DSWPD and moderate-to-severe DSWPD was made using the χ^2^ test for categorical variables and the Mann–Whitney *U*-test for the following continuous variables:”

The authors apologize for these errors and state that this does not change the scientific conclusions of the article in any way. The original article has been updated.

